# Semaphorin 4D is upregulated in neurons of diseased brains and triggers astrocyte reactivity

**DOI:** 10.1186/s12974-022-02509-8

**Published:** 2022-08-06

**Authors:** Elizabeth E. Evans, Vikas Mishra, Crystal Mallow, Elaine M. Gersz, Leslie Balch, Alan Howell, Christine Reilly, Ernest S. Smith, Terrence L. Fisher, Maurice Zauderer

**Affiliations:** 1 Vaccinex, Inc., Research, Rochester, NY USA; 2 Department of Neurology, Center for Health and Technology (SMD), University of Rochester Medical Center, Rochester, NY USA

**Keywords:** Semaphorin, Neurodegeneration, Reactive astrocytes, Huntington’s disease, Alzheimer’s disease, Metabolic reprogramming, Neurotransmitter recycling, Disease models, Pathogenesis

## Abstract

**Background:**

The close interaction and interdependence of astrocytes and neurons allows for the possibility that astrocyte dysfunction contributes to and amplifies neurodegenerative pathology. Molecular pathways that trigger reactive astrocytes may represent important targets to preserve normal homeostatic maintenance and modify disease progression.

**Methods:**

Semaphorin 4D (SEMA4D) expression in the context of disease-associated neuropathology was assessed in postmortem brain sections of patients with Huntington’s (HD) and Alzheimer’s disease (AD), as well as in mouse models of HD (zQ175) and AD (CVN; APPSwDI/NOS2^−/−^) by immunohistochemistry. Effects of SEMA4D antibody blockade were assessed in purified astrocyte cultures and in the CVN mouse AD model. CVN mice were treated weekly from 26 to 38 weeks of age; thereafter mice underwent cognitive assessment and brains were collected for histopathology.

**Results:**

We report here that SEMA4D is upregulated in neurons during progression of neurodegenerative diseases and is a trigger of reactive astrocytes. Evidence of reactive astrocytes in close proximity to neurons expressing SEMA4D is detected in brain sections of patients and mouse models of HD and AD. We further report that SEMA4D-blockade prevents characteristic loss of GABAergic synapses and restores spatial memory and learning in CVN mice, a disease model that appears to reproduce many features of AD-like pathology including neuroinflammation. In vitro mechanistic studies demonstrate that astrocytes express cognate receptors for SEMA4D and that ligand binding triggers morphological variations, and changes in expression of key membrane receptors and enzymes characteristic of reactive astrocytes. These changes include reductions in EAAT-2 glutamate transporter and glutamine synthetase, key enzymes in neurotransmitter recycling, as well as reduced GLUT-1 glucose and MCT-4 lactate transporters, that allow astrocytes to couple energy metabolism with synaptic activity. Antibody blockade of SEMA4D prevented these changes and reversed functional deficits in glucose uptake.

**Conclusions:**

Collectively, these results suggest that SEMA4D blockade may ameliorate disease pathology by preserving normal astrocyte function and reducing the negative consequences of reactive astrogliosis.

**Supplementary Information:**

The online version contains supplementary material available at 10.1186/s12974-022-02509-8.

## Background

Clinical manifestations of HD and AD clearly indicate neuronal deficits, however, the loss of glial homeostatic functions and the intimate connection and interdependence of astrocytes and neurons suggests that astrocytes may contribute to neuronal pathology by initiating [1] and/or amplifying neuronal dysfunction. Reactive glia have been increasingly recognized as key contributors to progression of AD, HD, PD, ALS and other slowly progressive neurodegenerative diseases. The role of astrocytes in neuropathogenesis is complex. Reactive astrocytes may be advantageous in some circumstances, but in response to chronic neuronal stress, they create a disease-permissive landscape that can contribute to neuronal dysfunction, degeneration and neurological deficits [1–4]. Astrocytes are a complex and heterogeneous cell population [5, 6] and there is increasing evidence for region-specific diversity [7, 8], which is not as yet fully described or understood. Yet, disease-associated changes with deleterious effects as well as reduced capacity to carry out normal supportive and protective functions suggests that astrocytes may contribute to the pathogenesis and progression of HD [4, 9, 10], AD [11] and other neuroinflammatory and neurodegenerative diseases [12, 13]. Triggers of astrocyte reactivity, therefore, represent rational targets for therapeutic intervention to restore beneficial homeostatic effects and reprogram downstream pathogenic effects.

Reactive astrogliosis is characterized by morphological and functional changes that can contribute to gain of inflammatory processes [14–17] as well as loss of normal homeostatic functions. Astrocytes play a critical role in the integration of neural synaptic networks and are well-positioned to couple energy metabolism with synaptic activity. Dysregulation of normal functions include impairments in glucose uptake and neurotransmitter recycling due to downregulation of key receptors and enzymes in the respective pathways, including GLUT-1, MCT-4, and glycolytic enzymes that regulate metabolic activity [18, 19], as well as EAAT-2 and glutamine synthetase (GS) required for recycling glutamate and GABA transmitters [20]. Upregulation of glial fibrillary acidic protein (GFAP) and cytoskeletal rearrangements that give rise to hypertrophic cell bodies with retracted short processes and loss of fine processes [15] are additional hallmarks of astrogliosis. These morphologic changes may also influence astrocyte pathogenesis via disruption of normal cell interactions that depend on cytoplasmic processes, including astro-neuronal and astro-endothelial contacts [21–24].

Semaphorin 4D (SEMA4D) was initially described as an axonal guidance molecule during neuronal development. In multiple cell types, including those of the immune, vascular, and nervous systems, SEMA4D binding to its plexin (PLXN) receptors (PLXN-B1 and -B2) signals through small membrane Rho GTPases [25–27] to regulate the actin cytoskeleton and to activate NfκB, a master regulator of inflammatory cytokines [28–31]. SEMA4D is biologically active both in a membrane-bound cellular form and as a proteolytically cleaved soluble mediator. We and others have previously reported that, in the CNS, SEMA4D (i) induces activation of microglia [32–34]; (ii) inhibits migration and differentiation of glial progenitor cells that can replace damaged oligodendrocytes and replenish astrocytes [26, 35, 36], and (iii) disrupts endothelial tight junctions that are required for the integrity of the blood–brain barrier (BBB) [25, 32]. Upregulation of SEMA4D following spinal cord injury [37, 38] has been reported, and interactions between SEMA4D and PLXN-B1 and -B2 receptors have been implicated in neuroinflammation and pathogenesis of experimental autoimmune encephalomyelitis [32, 39]. Astrocytes have been reported to express abundant levels of the high affinity PLXN-B1 receptor in brain of AD patients [40]. However, the sources of SEMA4D ligand in brain, as well as its possible role in triggering reactive astrocytes and their contribution to neurodegenerative processes have not been fully described. Here, we characterize SEMA4D expression in neurodegenerative disease settings of HD and AD, and report SEMA4D-induced changes in cytoskeletal morphology and regulation of key receptors and enzymes required for normal astrocytic functions in glucose metabolism and neurotransmitter recycling.

We previously reported a role for SEMA4D in a preclinical model of HD. PLXN receptors are expressed in GFAP+ striatal astrocytes in YAC128 HD transgenic mice. In addition, treatment with an anti-SEMA4D antibody reduced anxiety-like behavior and cognitive deficits, as evidenced by rescue of spatial memory and learning, along with significant reduction in brain atrophy and preservation of healthy medium spiny neurons, as indicated by DARPP-32 immunoreactivity in this model [41]. The present work reports an advancement in our knowledge of SEMA4D expression and correlated astrocytic changes in murine models of HD and AD and evaluates SEMA4D expression and its cellular distribution in sections of staged HD patient brain autopsy samples. In order to investigate whether this pathogenic mechanism may be more broadly relevant to slowly progressive neuroinflammatory/neurodegenerative diseases, we here extend the analysis of SEMA4D expression, astrocyte morphology and regulation of GS expression to human autopsy sections from AD brain. In addition, we investigate pathogenic and functional effects of SEMA4D antibody blockade in the CVN (APPSwDI/mNos2^−/−^) transgenic mouse model of AD.

These studies indicate that SEMA4D is sparsely expressed in normal brain, but is upregulated in neurons during disease progression in both mouse models and human disease. Increased expression of SEMA4D ligand was associated with changes in astrocyte morphology and downregulation of GS expression, a previously described marker of reactive astrocytes [20] that is associated with impairment of neurotransmitter recycling, a key astrocytic function. To further characterize astrocyte receptor-mediated changes triggered by SEMA4D, we investigated the effects of SEMA4D in a series of studies using purified human astrocyte cultures to evaluate whether SEMA4D triggers receptor-mediated morphological and molecular changes and loss of homeostatic functions that are characteristic of reactive astrocytes and impair their ability to support normal neuronal functions. Results suggest that blockade of SEMA4D signaling may serve to normalize astrocytic functions and slow astrocyte reactivity that is believed to contribute to pathology and symptoms of neurodegenerative disease.

## Methods

### Study design

The objectives of the study were to characterize expression of SEMA4D in diseased brain and to investigate potential role of ligand signaling to PLXN-B1/-B2+ astrocytes in neurodegenerative diseases that shared a common reactive pathology. Using controlled laboratory experiments, expression of SEMA4D and astrocyte reactivity was evaluated using IHC in brain regions relevant to respective disease from patients and mouse models of HD and AD. To evaluate role of SEMA4D in vivo, we evaluated treatment effects of SEMA4D antibody blockade in transgenic CVN mice using IHC histopathology as well as behavioral assessments using radial arm water maze. In vivo experimentation was conducted in a blinded manner (see Supplemental Methods). To further investigate receptor-mediated effects on astrocytes, in vitro assays using purified astrocyte cultures from two species, human and rat, and two vendors for each species were evaluated to determine consistent and reproducible effects. All data were collected and processed blinded to treatment.

### Immunohistochemistry: mouse brain sections

Whole mouse brains from heterozygote (HET) zQ175 knock-in (KI) (CHDI-81003003) Huntington’s mice and wild-type (WT) littermate controls; collected at ~ 3, 6, 9 months of age were obtained from CRL Discovery Services. Q175 knock-in mice express the mHTT allele with the expanded CAG repeats within the native mouse huntingtin gene [42]. Formalin-fixed mouse brains were first coronally dissected (anterior to posterior) at approximately; section 1, from anterior to 1.33 mm ant. ac, section 2, 1.33 mm ant. ac to − 1.67 mm ant. ac, section 3, − 1.67 mm ant. ac to − 4.67 mm ant. ac and section 4 from − 4.67 mm ant. ac to posteriorly. The four major coronal planes were aligned and paraffin embedded with the posterior position pointing upwards. Three successive 5-µm-thick, 25-µm apart coronal sections with coronal representation from four levels, anterior to posterior, were employed for immunohistological examination. See below for preparation of tissue sections from CVN studies. Immunofluorescence staining of murine tissue sections were performed for mouse semaphorin 4D (PA5-47711, Invitrogen), NeuN (ab177487, Abcam), GFAP (ab7260, Abcam), somatostatin (H-106, Santa Cruz), Neuropeptide-Y (NPY; NBP-19808, Novus), synaptophysin (101004, SySy), VGLUT-1 (TA317309, Orogene), Olig-2 (ab109186. Abcam), in accordance with manufacturer-recommended concentrations combining host-dependent secondary Alexa Fluor antibodies. For all mouse tissues, the resulting images were mapped with mouse brain atlas to generate ROI and discretion was maintained to avoid regional overlap.

### Human brain sections

Human HD brain specimens were obtained from National Institute of Health, Neurobiobank (NBB) Brain and Tissue Repositories (protocol number-1105-HDPilotSep2018) through participating brain banks of The Mount Sinai/JJ Peters VA Medical Center NIH Brain and Tissue Repository, Icahn School of Medicine at Mount Sinai; Harvard Brain Tissue Resource Center, Harvard Medical School; University of Miami Brain Endowment Bank, Miller School of Medicine, University of Miami and Brain and Tissue Bank, School of Medicine, University of Maryland. Samples of human frontal cortex regions were obtained from frontal cortex (inferior frontal gyrus including Brodmann area-BA 44–45) and parietal lobe (somatosensory cortex, including Brodmann area-BA 1, 2, 3 and part of frontal cortex BA4), and striatum (caudate/putamen). Age (years) and gender characteristics (F = female, M = male) of samples used in this study are as follows: Normal controls: 63 (M), 56 (F), 54 (M); HD Stage 0: 81 (F), 49 (M), 52 (F); HD Stage 1: 74 (M), 69 (M), 57 (M); HD Stage 2: 63 (F), 65 (M), 55 (F). FFPE blocks containing roughly 25 × 15 × 5 mm^3^ size of brain were processed for 3–4 consecutive 16-µm thin sections for immunohistochemistry.

Human AD brain specimens were obtained from BioChain Institute, Inc. Newark, CA. The tissue included areas from frontal, temporal, parietal, occipital lobes and corpus callosum; from diencephalic region, the thalamus and from metencephalonic structure, the pons and cerebellum. Quantification was determined from areas of thalamus, temporal lobe (Brodmann area-BA 38), and frontal cortex (Inferior frontal gyrus, Brodmann area-BA 44–45). Age (years) and gender characteristics (F = female, M = male) of samples used in this study are as follows: normal controls: 89 (M), 68 (M), 60 (M), 50 (F), 54 (M), 54 (F); AD subjects: 73 (M), 73 (M), 87 (M), 88 (M), 85 (F). 5 µm FFPE sections containing roughly 8 × 10 mm size of brain, were processed for immunohistochemistry.

Immunofluorescence staining of human tissue sections were performed for human semaphorin 4D (ab134128, Abcam), glutamine synthetase (MA5-27749, Invitrogen), HuC/HuD (A-21271, Invitrogen) and GFAP (ab7260, Abcam) in accordance with manufacturer-recommended concentrations combining host-dependent secondary Alexa Fluor antibodies.

### Microscopy

Imaging of stained tissue sections were performed on AxioObserver7 Automated Inverted Microscope System with EC Plan-Neofluar × 10/0.30 and Objective Plan-Apochromat ×20/0.8. For immunofluorescence intensity and cell numbers, whole stained tissue in each slide, three consecutive slices per subject were analyzed with Fiji/I3mage J software (National Institutes of Health, Bethesda, MD, http://imagej.nih.gov/ij/). Relative levels of fluorescence intensity were calculated as the sum of the integrated density for each fluorescence stain image with default segmentation divided by the area, where data are supplied as average integrated fluorescent intensity. Mean of the sum of integrated fluorescence intensity of 3 consecutive sections + SEM from each individual is shown for each subject/condition. No staining was observed in negative controls with respective isotype control primary antibodies.

### Fractal dimensions

For astrocytes fractal dimensions analysis, 5-µm-thick brain coronal sections were imaged on AxioObserver7 Automated Inverted Microscope with Plan-Apochromat × 20/0.8; × 40/0.95 objectives and Axiocam 702 Monochrome Camera. Images were taken in different optical planes in order to resolve the finest details possible at this resolution. Based on the extended depth of field algorithm, Zen 3.0 (Carl Zeiss Microscopy, GmbH, 2019) generated a final image which recapitulated all the details of the cell. Cells without visible nuclei were rejected in this study. Fractal dimension analysis was performed on GFAP+ astrocytes (*n* ~ 500 to 900 astrocytes/sample) for each section for each age group (cases/controls). Fractal analysis was performed as described previously [43, 44] using Fiji/Image J software (National Institutes of Health, Bethesda, MD, http://imagej.nih.gov/ij/) along with the FracLac plug-in (A. Karperien—Charles Sturt University, Australia). Fractal dimensions (FDs) were calculated by the box-counting algorithm as the slope of the regression line for the log–log plot of the scanning box size and the count from a box-counting scan.

### Treatment antibodies for in vivo and cell culture

Anti-SEMA4D monoclonal antibodies (MAb) were generated in our laboratory as described in Fisher et al. [45]. For in vivo treatment, anti-SEMA4D MAb67, mouse IgG1 was utilized. Anti-human CD20 clone 2B8.1E7 hybridoma was obtained from the American Type Culture Collection (ATCC). 2B8.1E7, which is specific for human CD20 and does not cross-react with murine B cells, was used as an irrelevant control IgG1, isotype-matched with MAb67. For in vivo treatment of CVN mice, mouse antibodies were purified from hybridoma supernatants using ProG affinity column and ion exchanges and were confirmed to have < 0.5 EU/mg endotoxin levels, > 95% purity, and < 5% high-molecular-weight species. For in vitro astrocyte assays, humanized anti-SEMA4D antibody VX15/2503 (pepinemab) was used with human IgG4 isotype control antibody (irrelevant antibody, anti-C35, Vaccinex), anti-PLXN-B1 (Mab37491, Mouse IgG1, R&D Systems), anti-PLXN-B2 (Mab5329, Mouse IgG2a, R&D Systems), isotype controls mouse IgG1 (Vaccinex, Mab C35), mouse IgG2a (Vaccinex, Mab7.16.4) were either produced internally or purchased from commercial source and further purified at Vaccinex to meet quality standards of < 0.5 EU/mg endotoxin levels.

### CVN mouse model

Homozygous APPSwDI/NOS2^−/−^ bigenic mice (CVN-AD) mice were produced by crossing mice expressing the vasculotropic Swedish K670N/M671L, Dutch E693Q, and Iowa D694N human APP mutations under control of the Thy-1 promoter with mNos2^−/−^ (B6 129P2Nos2tau1Lau/J) mice (Charles River Laboratories). Male and female wild type and CVN-AD mice were treated in a gender-balanced and blinded manner with anti-SEMA4D (MAb67) antibody or mouse IgG1 isotype control (MAb 2B8) (30 mg/kg, IV, weekly × 12) at 26 weeks of age. The mice were subjected to baseline behavioral testing at age weeks 10–12 and mice reaching the criteria were included in the follow-up (*n* = 10–13 in each experimental group). Radial arm water maze (RAWM) testing was again performed at 39 weeks of age. In vivo experimentation was conducted in a blinded manner. For instance, the individual dosing the mice is different from the individual actually running the phenotypic tests. Alternatively, if the same person will do dosing and phenotypic testing, that individual does not have the code for mice receiving drug or vehicle and the vials with vehicle or drug are labeled so as not to allow distinction. Mice were allowed at least 30 min acclimation to the experimental room conditions prior to testing. Two-day radial-arm water maze has been described in detail previously [46]. Briefly, a six-arm maze was submerged in a pool of water, and a platform was placed at the end of one arm. The mouse received 15 trials per day for 2 days and each trial was started from a different arm, while the arm containing the platform remained the same for each trial. The first 11 trials alternated between the visible and hidden platform. The final 4 trials for day 1 and all trials on day 2 were tested with a hidden platform. Each block represents average of 3 trials (5 blocks/day). The number of errors (incorrect arm entries) were counted over a 1 min period, and the latency to find the platform was recorded. The errors and latency scores were averaged for each block (consisting of three trials) resulting in 10 blocks for the 2-day period. In all trials, platform finding success, latency, and abnormal swimming performance if any were recorded; animals showing poor platform finding success or swimming performance were excluded from analysis. Two-way ANOVA repeated measures was utilized to determine group differences over the 10 blocks. Live animal phase was conducted according to the following guidelines: Act on Use of Animals for Experimental Purposes (62/2006), Finland; Ordinance number 36/EEO/2006 on experimental animals, Ministry of Agriculture and Forestry, Finland; Directive 2010/63/EU of the European Parliament and of the Council of 22 September 2010 on the protection of animals used for scientific purpose. All animal experiments are approved by the State Provincial Office of Southern Finland. Brains were harvested at 41 weeks of age, perfused with non-heparinized saline and post-fixed by immersion in 4% PFA for IHC analysis. Analysis focused on hippocampal regions including CA1, dentate gyrus and subiculum—NPY, V-GLUT and synaptophysin were quantified in dentate gyrus region and somatostatin was quantified in subiculum. Percentage of somatostatin-positive signal was quantified within the subiculum of all animals and normalized to total subiculum area scanned and percentage of NPY-positive signal was quantified within the dentate gyrus of all animals and normalized to total dentate gyrus area scanned.

### In vitro human astrocyte studies

Normal non-immortalized primary human astrocytes (CC-2565, Lonza) were acquired at passage two, and cultured for a maximum of 3–4 additional passages in astrocyte basal growth media (CC-3186 ABM™, Lonza) with 2% v/v FBS and astrocyte growth medium supplements (AGM; Lonza Inc., Anaheim, CA, USA), including 5 mL l-glutamine, 0.50 mL ascorbic acid, 0.50 mL hEGF 0.50 mL, and Insulin 1.25 mL. Prior to culture initiation, standard tissue grade polystyrene culture flasks were coated with poly-d-lysine (P6407, Sigma-Aldrich) substrate for optimum astrocyte growth and differentiation. Tissue origin of normal human astrocytes was cerebral cortex (grey matter). Data were reproducible using a second source of human primary astrocytes from Neuromics, Edina (HNC001). Purity of cultures was assessed by GFAP stain (> 95% GFAP-positive cells) and visual inspection of cell morphology and growth characteristics. Astrocytes were seeded on #1.5; 0.16 to 0.19 mm cover glasses in 24 well plates at a seeding density of 5000 cells/cm^2^. At around 75% visual confluency, the cells were treated for 24 or 48 h with Control protein rEGFR-his (5 µg/mL, a his-tagged protein that was produced/purified under similar conditions and does not bind to astrocytes) or rSEMA4D-his (5 µg/mL), combination treatments with antibodies to SEMA4D, PLXN-B1, PLXN-B2, or irrelevant isotype control antibodies (25 µg/mL). Each condition was assessed in 6 replicate wells. Immunofluorescence staining of fixed cells (acetone:methanol; 1:1) were performed for semaphorin 4D (ab134128, Abcam), EAAT-2 (711020, Invitrogen), Glut1 (ab15309, Abcam), and MCT4 (ab244385, Abcam) in accordance to manufacturer recommendation. Imaging of stained cover glasses were performed on AxioObserver7 Automated Inverted Microscope System with EC Plan-Neofluar × 10/0.30 and Objective Plan-Apochromat × 40/0.95. For immunofluorescence intensity, whole stained cover glasses were evaluated for each condition, each coverslip was analyzed by dividing into three quadrants to avoid corners, mean of each quadrant was calculated for each replicate well.and analyzed with Fiji/Image J software (National Institutes of Health, Bethesda, MD, http://imagej.nih.gov/ij/). The morphometric characterization of astrocytes in in vitro conditions were done on immunofluorescence GFAP and DAPI 40X images acquired by Zeiss AxioObserver imaging system, with imaging configuration as Plan-apo × 63/1.40 oil, DIC M27, Apotome, and with the aid of AutoNeuriteJ, an imageJ/Fiji plugin for Simple Neurite Tracer (Fiji-ImageJ, NIH, Plugins-Segmentation-Simple Neurite Tracer; by Mark Longhair and Tiago Ferreira, MRC and Janelia Campus; http://imagej.net/Simple_Neurite_Tracer; with default segmentation sigma, 0.196) that can measure and classify complex cell structures including length and number of cell processes [47]. The resulting images were 3D-reconstructed and identifiable cell processes length and branch numbers were semi-automatically quantified with Simple Neurite Tracer. For each treatment condition, length and number of primary processes are quantified from > 500 individual astrocytes/coverslip with clear contained DAPI stain and non-truncated main process, 3 wells were randomly picked from each of the 6-well replicates.

For the glucose uptake assay, cells then were washed twice with HBS before treatment. Glucose detection was performed using Glucose-Glo™ Assay (J6022, Promega) according to the manufacturer's recommendations. Buffer-only control was used as negative control to measure background signal, data have been presented as normalize fluorescence units (AU) after calculating signal-to-background ratios.

### Statistical analysis

Statistical analysis of IHC data was performed with IBM SPSS software (version 1.0.0.1327); one-way ANOVA was used to compare multiple (> 2) groups with one independent variable followed by Tukey’s multiple comparison. Data outlier’s and normal data distributions were assessed through boxplot and Shapiro–Wilk test. Homogeneity of variances between groups within each dataset was determined with Levene's test. Statistical significance was determined with one-way ANOVA for *p*-values < 0.05 with Tukey's post hoc test. Dataset with violations in homogeneity of variances, statistical significance was determined with Welch one-way ANOVA for *p*-values < 0.05 with Games–Howell post hoc analysis.

Two-way ANOVA was used to compare multiple (> 2) groups with two or more independent variables with 95% confidence intervals and Bonferroni-adjustment. In vitro treatment studies were analyzed with double multivariate MANOVA or multiple regression to determine differences with treatment type and with one-way multivariate analysis to incorporate dependent variables effects with treatment condition. For multivariate analysis, normality violations were assessed by Shapiro–Wilk's test, multicollinearity with Pearson correlation and multicollinearity analysis with Mahalanobis distance. The significance levels are reported with Tukey post hoc tests for multivariate analysis. With violations in normal distribution of data, group differences and statistical significance were determined using Kruskal–Wallis *H* test. The distribution differences among dependent variables between different groups were assessed by visual inspection of boxplots and median rank score was used for data with comparable distribution, whereas mean rank for data with dissimilar distribution. A pairwise comparison was performed using Dunn's procedure with a Bonferroni correction for multiple comparisons. All error bars in figures represent the standard error of the mean (SEM). *p < 0.05; **p < 0.01; ***p < 0.001; ****p < 0.0001.

## Results

### Upregulation of SEMA4D and reactive astrocytes during Huntington’s disease progression

To characterize SEMA4D expression and its cellular distribution during HD progression, we evaluated tissues representing early, mid, and late stage disease in the Q175 KI mouse model of HD, characterized by progressive disease reflective of adult-onset HD with reported astrocyte dysfunction [48] and cytoskeletal changes [9]. Mouse brain coronal sections were evaluated using immunohistochemical staining to colocalize SEMA4D with various cell-specific markers for neurons (NeuN), astrocytes (GFAP, GS) and oligodendrocytes (Olig2). Three heterozygous zQ175 KI mice and wild type controls from each age group at 3, 6, and 9.3 months of age were employed for these investigations.

**Fig. 1:**
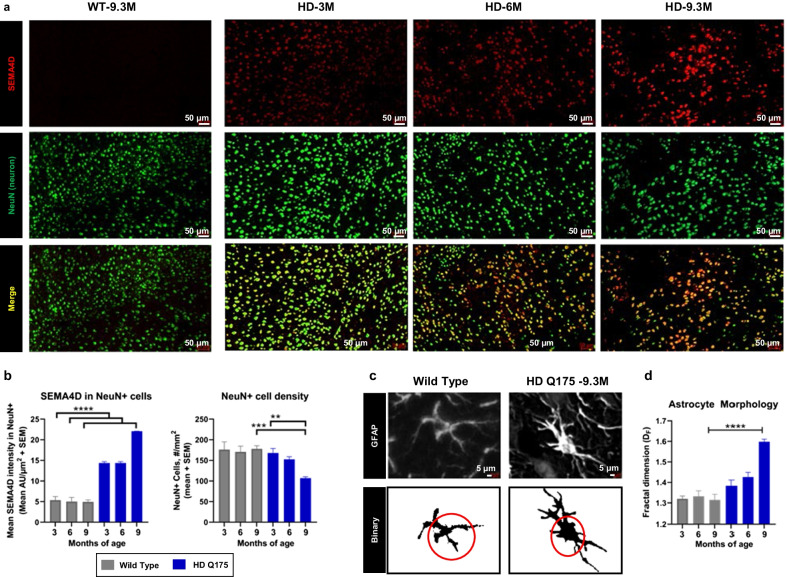
SEMA4D is progressively upregulated in HD neurons in parallel with evidence of astrocyte activation in Q175 KI HD mice. **a** Q175 knock-in mouse model of HD exhibits age-dependent upregulation and colocalization of SEMA4D in cortical neurons. **b** Quantification of SEMA4D expression and number of NeuN+ neurons in Q175 model with three mice/age group. Quantification analysis was performed on the entire coronal section of each mouse; mean + SEM of the sum of integrated fluorescence intensity/mouse/age group is shown. **c** Representative images of GFAP staining of caudoputamen at × 20 magnification. **d** Fractal dimension analysis of GFAP+ astrocytes in mouse frontal cortex were obtained and quantified from coronal sections (~ 5 µM); significant changes in brains of Q175 mice at 9.3 months of age observed, where data are presented as mean of all astrocytes + SEM. Group differences and statistical significance were determined using two-way ANOVA, where significance is reported at 95% confidence intervals and *p*-values are Bonferroni-adjusted and are indicated by *p < 0.05, **p < 0.01, ***p < 0.001, ****p < 0.0001

SEMA4D levels were very low in adult normal brain tissue but found to be increased in neurons of HD KI Q175 mice (Fig. 1a, b); levels increasing with age as the disease progressed. Upregulation of SEMA4D can be detected even at 3 months of age, when some psychological and cognitive changes become evident. A significant reduction in the density of NeuN+ neurons at 9 months of age was observed (Fig. 1b). SEMA4D was also detected at relatively lower levels on some Olig2+ cells (Additional File 1: Fig S1) but rarely on GFAP+ cells.

We have previously reported that SEMA4D triggers depolymerization of the actin cytoskeleton in glial progenitor cells [32]. Similar cytoskeletal changes, together with significant changes in gene expression and function, are characteristic of reactive astrocytes [49, 50]. To characterize and quantify complex morphological changes in heterogeneous astrocytes, high resolution multiplex apochromat imaging on various planes of brain tissue sections coupled with scale-invariant parameters like fractal dimensions (Df) [50, 51] were employed to measure spatial occupation and complexity. Df analysis has been used to effectively differentiate between fibrous, protoplasmatic and activated astroglia relevant to disease [50]. Astrocytes in HD KI Q175 mice (Fig. 1c) display characteristic high GFAP immunoreactivity, enlarged cell soma, and significant increase in Df values compared to wild-type mice at 9 months of age (Fig. 1d).

**Fig. 2:**
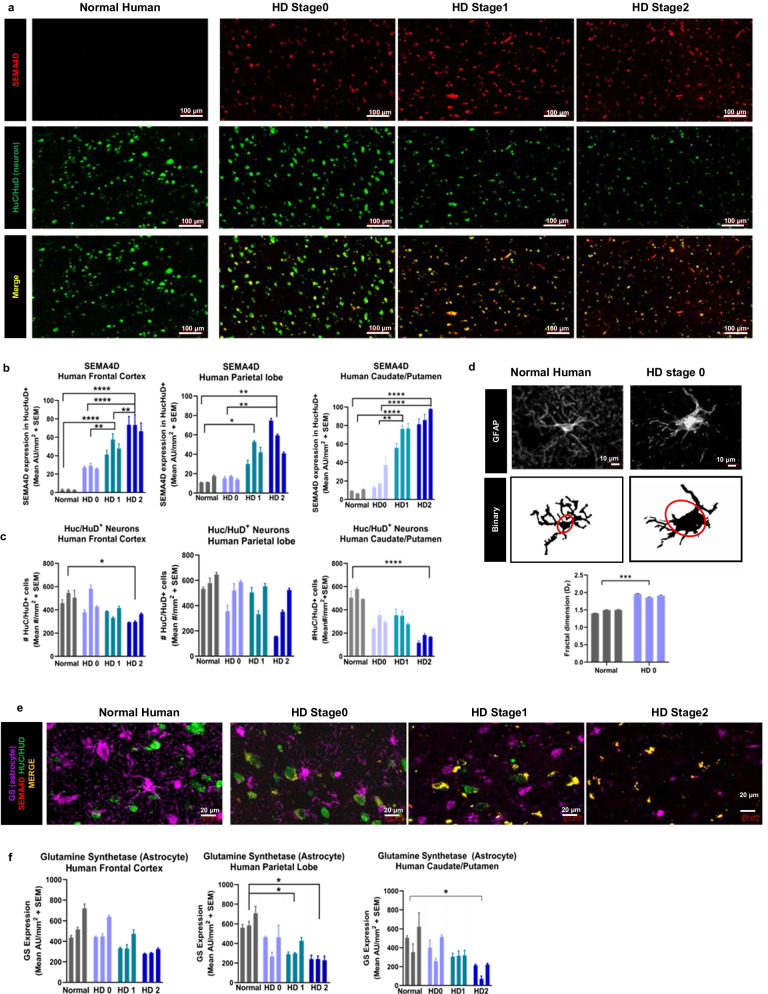
SEMA4D is increasingly upregulated in neurons during human HD disease progression; changes in astrocyte GS expression and morphology are indicative of astrogliosis. Human brain autopsy sections from non-diseased “normal” human and HD pathological stage 0, 1, and 2 were processed for SEMA4D, Huc/HuD (neuronal cell body), GFAP (astrocytes), and GS (astrocyte cell body and processes). Brain regions included frontal cortex, parietal lobe, and striatum (caudate/putamen). **a** Representative images of frontal lobe illustrate upregulation of neuronally expressed SEMA4D (100-micron scale shown). Quantification of **b** SEMA4D expression in HucHUD+ cells and **c** HucHUD+ neuron density. **d** GFAP staining of frontal lobe are shown; soma is circled in binary image conversion. Fractal dimension analysis of GFAP+ astrocytes demonstrate significant changes between non-diseased and HD. Group differences and statistical significance was determined by an independent-samples *t*-test. **e** GS expression is reduced and retracted astrocytic endfeet are observed in HD, compared to normal. (20-micron scale shown). **f** Quantification of GS in GFAP+ cells. Each bar represents autopsy tissue from one individual; mean of the sum of integrated fluorescence intensity of 3 consecutive sections/individual + SEM is shown for each subject/condition. Group differences and statistical significance was determined using one-way ANOVA with Tukey post hoc analysis

We sought to extend these findings to human disease and further characterize SEMA4D expression and reactive astrocytes using multivariate assessments in different human brain regions. Towards that end, we examined autopsy samples obtained from the NIH brain bank (NBB#1105) of 3 subjects each representative of normal control, and HD pathological stage 0, stage 1, and stage 2. For each subject, analysis was repeated on each of 3 consecutive sections and shown as an average of the 3 sections for that subject. Staining for SEMA4D and the neuronal marker HuC/HuD (Fig. 2a) shows statistically significant upregulation of SEMA4D in neurons (Fig. 2b) and evidence of neuronal loss (Fig. 2c) in frontal and parietal lobes, and in the caudoputamen striatal regions that were examined. Early neuronal loss, especially of medium spiny neurons in the striatum with increasing involvement of the cortical regions as the disease progresses, is characteristic of human HD [52]. SEMA4D levels in HuC/HuD+ neurons continue to increase with increasing pathological stage, perhaps indicating increasing neuronal stress during disease progression.

Astrocyte reactivity was evaluated in human tissues using multivariate assessments of (1) morphology, (2) GFAP immunoreactivity, and (3) expression of GS, a key enzyme required for recycling neurotransmitters, that is highly expressed in normal astrocytes but downregulated in reactive astrocytes [20]. As observed in mouse brains, high GFAP immunoreactivity, enlarged soma size and a significant difference in Df measurements are observed in HD brain tissues relative to normal human controls (Fig. 2d). In addition to increased GFAP expression and morphologic alterations characteristic of cytoskeletal rearrangements, GS enzyme levels were evaluated to visualize morphologic changes in cytoplasmic processes, as well as functional changes related to neurotransmitter recycling in human tissues. The close proximity of GS-positive cytoplasmic processes of stained GFAP+ astrocytes (purple) to HuC/HuD-positive neurons (green, turning to yellow with SEMA4D co-stain) observed in normal healthy brain tissue appears to be disrupted in the HD brain (Fig. 2e). Furthermore, reduction of GS expression starting at HD stage 0 indicates the presence of reactive astrocytes early in human disease (Fig. 2f). Collectively, increased GFAP expression, morphological changes and downregulation of GS are consistent with a reactive astrocyte phenotype and coincident with HD disease progression and increasing upregulation of SEMA4D in neurons.

### Upregulation of SEMA4D and astrocyte reactivity in human AD brains

**Fig. 3:**
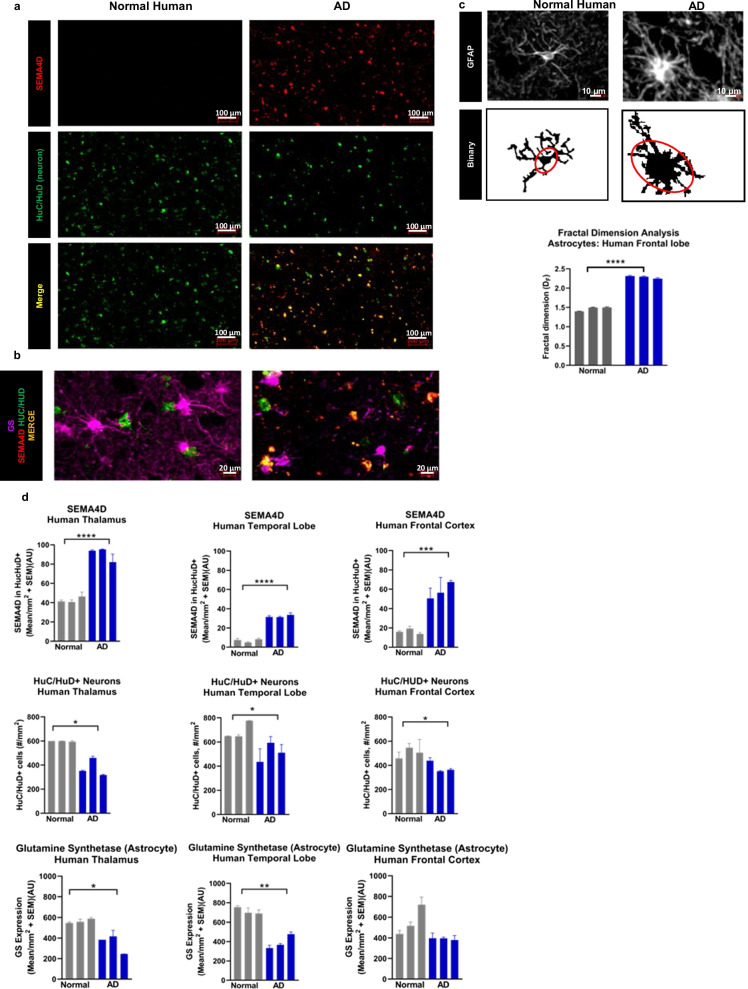
SEMA4D is upregulated in neurons of human AD; changes in astrocyte GS expression and morphology are indicative of astrogliosis. Human brain autopsy sections from non-diseased human and AD, with representations from thalamus, temporal lobe, and frontal cortex were processed for SEMA4D, Huc/HuD (neuronal cell body), GFAP (astrocytes), and GS (astrocyte cell body and processes). Representative images of human temporal lobe sections stained for **a** SEMA4D and Huc/HuD and **b** GS, and **c** GFAP staining; soma is circled in binary image conversion. Fractal dimension analysis of GFAP+ astrocytes demonstrate significant changes between non-diseased (“normal”) and AD individuals. (Note different scales shown in **a**–**c**) **d** Quantification of SEMA4D in HuC/HuD+ cells, HuC/HUD+ cell density, and GS expression in GFAP+ cells. Each bar represents autopsy tissue from one individual; mean of the sum of integrated fluorescence intensity of 3 consecutive sections/individual + SEM is shown for each subject/condition. Group differences and statistical significance was determined using one-way ANOVA with Tukey post hoc analysis

To determine if SEMA4D upregulation is common to underlying pathology in another neurodegenerative disease in addition to HD, we evaluated postmortem brain sections from subjects with AD. Consistent with observations in HD, we observed significant upregulation of SEMA4D and a reduction in neuronal density in AD brains compared to normal controls (Fig. 3a). Additionally, we observed significant reduction in GS expression (Fig. 3b), high GFAP immunoreactivity, and morphologic changes including enlarged cell soma and significant increase in Df values associated with reactive astrocytes in AD brains (Fig. 3c). Significant changes in multiple regions of interest including the frontal cortex, temporal lobe and thalamus of AD affected subjects are shown in Fig. 3d.

### SEMA4D blockade inhibits reactive astrocytes and restores neuronal and cognitive deficits in mouse model of AD

Considering that expression of SEMA4D is upregulated in disease along with reactive astrocytes, which have been reported to contribute to disease progression of both HD and AD, as well as our previously reported therapeutic activity of a SEMA4D blocking antibody to ameliorate neurodegenerative processes in a KI mouse model of HD [41], we sought to determine if SEMA4D blockade can also modulate behavior and neuropathology in an AD transgenic model. The CVN mouse model displays characteristics of human AD progression, including reactive astrocytes, neuroinflammation, amyloid deposition, phosphorylated tau protein, spatial memory impairments, and significant neuronal death in hippocampal, cortical, and thalamic regions [53–56]. As discussed below, others have reported that the background APP variant expression in this model is associated with a molecular subtype of AD that may be driven by inflammatory processes [57]. This model allowed us to precisely investigate SEMA4D-dependent effects on astrocyte reactivity, as well as the accompanying neuropathologic alterations and behavioral symptoms.

**Fig. 4:**
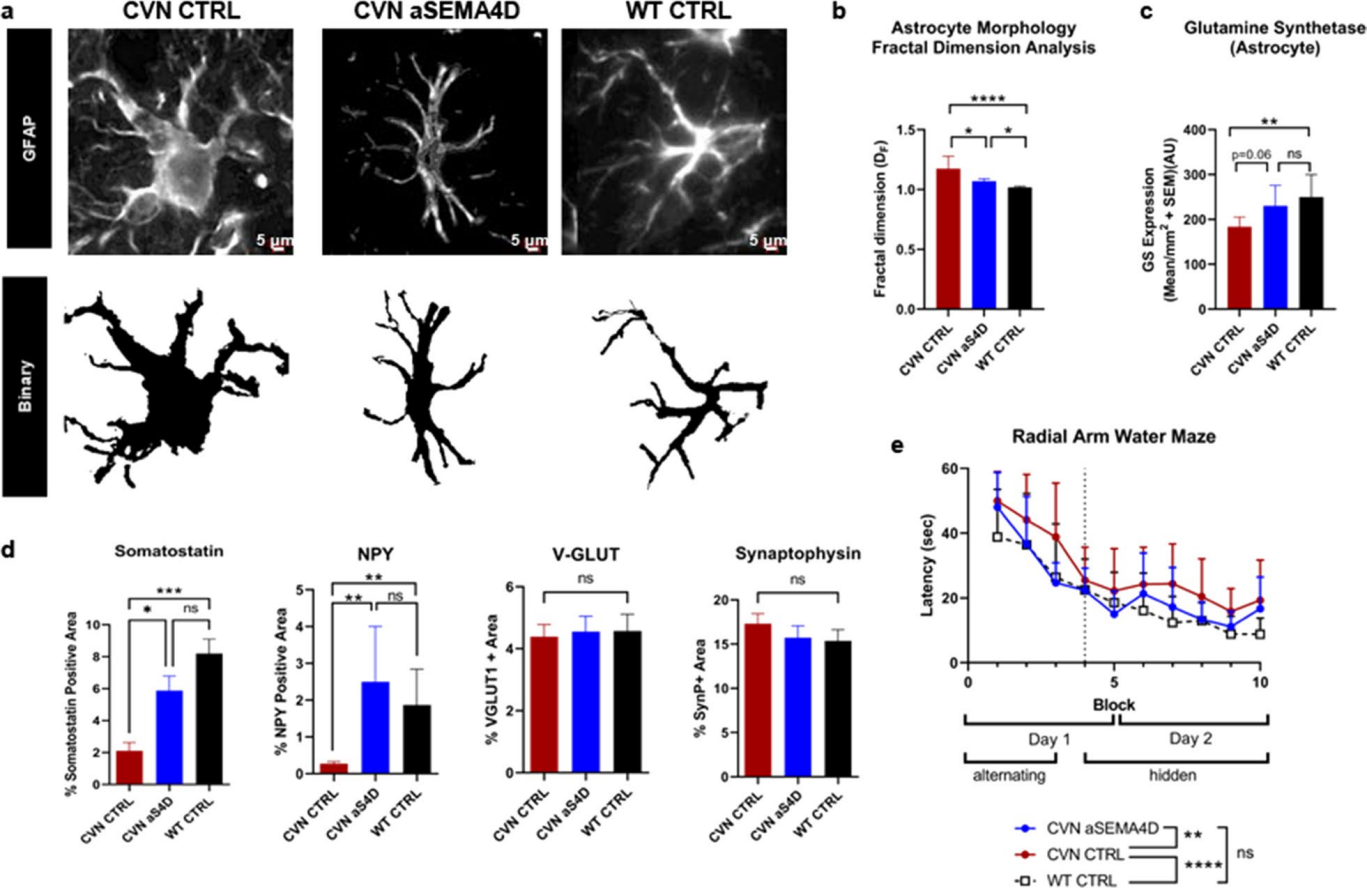
SEMA4D antibody treatment inhibits reactive astrocytes and restores neuronal and cognitive deficits in CVN mouse model of AD. CVN and WT mice were treated in vivo with anti-SEMA4D “aS4D” or mouse IgG1 isotype control antibody “CTRL”. **a** CA1 hippocampal region of CVN and wild type mice were stained GFAP and **b** fractal dimension analysis and **c** GS expression in GFAP+ cells demonstrates significant changes in brains of CVN mice compared to wild type, which is restored following treatment with anti-SEMA4D antibody. **d** The hippocampal region was stained with anti-somatostatin antibody or anti-Neuropeptide-Y (NPY) to identify specific subsets of inhibitory neurons. No effects on excitatory synapses were observed in diseased mice (as determined by Synaptophysin and VGLUT-1 staining). Percentages were quantified for all animals (*n* = 9–13/group) and normalized to total area scanned; group mean + SEM are shown. For **b**–**d**, group differences and statistical significance were determined using Kruskal–Wallis *H* test and subsequent pairwise comparisons were performed using Dunn's procedure with a Bonferroni correction for multiple comparisons. **e** Latency was assessed in Radial Arm Water Maze at week 36. Group means of all animals + SEM for each trial block is shown. Statistical significance was determined by 2way mixed ANOVA with repeated measures

Mice were treated weekly starting at 26 weeks of age, around the time of symptom onset, and brains were collected at 41 weeks of age, when major pathologic changes including glial activation and neuronal degeneration have occurred. SEMA4D upregulation and presence of reactive astrocytes was confirmed in the CVN mice. Morphologic alterations, including enlarged soma, short and thick branches, and significant changes in fractal dimensions analysis, as well as increased GFAP immunoreactivity were observed in CVN compared to WT mice (Fig. 4a–c). Treatment with SEMA4D blocking antibody preserved normal astrocyte morphology in hippocampal astrocytes of CVN mice (Fig. 4a, b). Additionally, expression of GS was significantly reduced in GFAP+ astrocytes from the hippocampus of CVN mice compared to wild type mice (*p* = 0.0054), while no significant difference in GS expression was observed in CVN mice treated with SEMA4D blocking antibody compared to wild-type controls (*p* = 0.9276) (Fig. 4c), indicating preservation of wild-type GS levels in CVN mice.

In addition to being a marker of reactive astrocytes, down regulation of GS, a cytoplasmic enzyme necessary for neurotransmitter function, has been reported to have a differential effect on loss of inhibitory GABAergic relative to excitatory glutamatergic signaling [20]. Loss of inhibitory synapses was observed in CVN mice, as evidenced by loss of both somatostatin and neuropeptide Y (NPY) immunoreactivity in the interneurons of the subiculum and dentate gyrus. In contrast, there was no difference observed in the number of total NeuN+ neurons (Additional File 2: Fig. S2a) and no effects on total or excitatory synapses in diseased as compared to normal control mice, as determined by synaptophysin and vesicular glutamate transporter 1 (VGLUT-1) expression (Fig. 4d).

Learning and spatial memory were assessed at 36 weeks of age using the radial arm water maze, in which latency to find a hidden platform was measured. CVN mice displayed increased latency compared to WT mice, but spatial learning was restored in mice treated with anti-SEMA4D antibody, as evidenced by improvement in latency comparable to WT mice (Fig. 4e). Similar trends in the number of errors were observed (Additional File 2: Fig S2b). These results demonstrate that SEMA4D blockade prevented the changes associated with reactive astrocytes and differential loss of inhibitory synapses characteristic of disease progression in CVN mice.

### SEMA4D triggers receptor-mediated astrocyte reactivity and reduced metabolic function

**Fig. 5:**
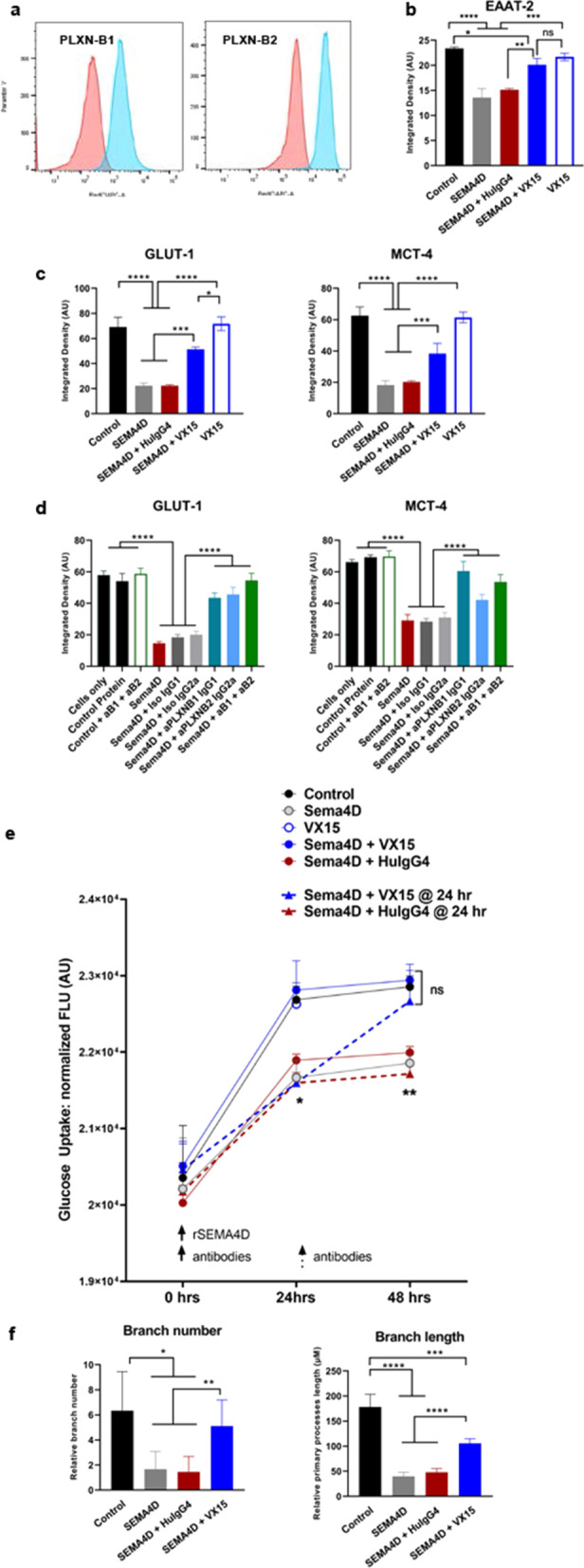
SEMA4D triggers receptor-mediated astrocyte reactivity, including changes in astrocyte morphology, expression of key transporters for glutamate recycling and energy metabolism and impairs astrocyte function of glucose uptake. **a** Primary human astrocyte cultures were stained for PLXN receptors (blue), compared to isotype control antibodies (red). Antibody blocking effect was determined by incubation with rSEMA4D or control protein, in presence/absence of anti-SEMA4D antibody/VX15 (human IgG4) or isotype-matched control antibodies for 48 h. Cultures were stained for **b** EAAT-2, and **c** GLUT-1 and MCT-4 transporters. **d** In a separate experiment, blocking of receptors was assessed using anti-PLXNB1 (mouse IgG1) and/or anti-PLXNB2 (mouse IgG2a) or isotype-matched control antibodies and the same conditions as above. Quantification is shown as; mean + SEM of replicates for each treatment. **e** Glucose uptake was measured in human astrocyte cultures treated as above. rSEMA4D was added at time 0 and antibodies were added at *t* = 0 (solid lines and circles) for inhibition or *t* = 24 h (dotted lines and triangles) to evaluate reversal of activity. Quantification for each condition is shown as average + SEM from 3 wells/condition/timepoint. **f** Morphologic changes showing length and number of primary processes. For **b** to **f**, multivariate regression analysis was performed to determine the effect treatment conditions and dependent variables with treatment type. The significance levels are reported with Tukey post hoc tests for multivariate analysis

To further investigate whether SEMA4D signaling to astrocytes can trigger astrocyte reactivity, we explored whether astrocytes express PLXN-B1 and -B2 receptors and if binding of SEMA4D impacts molecular, functional and morphologic changes in purified human astrocyte cultures [17]. Astrocyte cultures were grown in low serum conditions (2% v/v) using media specifically formulated for astrocytes and confirmed to predominantly contain astrocytes by > 95% of the cells expressing GFAP (Additional File 3: Fig. S3a). As shown in Fig. 5a, PLXN-B1 and -B2 receptors are highly expressed in purified primary human astrocyte cultures. Similar results were obtained in the presence or absence of serum.

Glutamate recycling and glucose transport are important normal functions of astrocytes and are reported to be significantly disrupted in reactive astrocytes [19, 58]. As discussed above, GS is an important enzyme for recycling of glutamate within the astrocytes, while excitatory amino acid transporter (EAAT)-2 is one of the major astrocytic transporters of glutamate, regulating glutamate uptake at the synaptic cleft in order to maintain homeostasis and potentially prevent adventitious signaling and excitotoxic activity. Binding of rSEMA4D to its receptors expressed on primary human astrocytes significantly reduced expression of EAAT-2 (Fig. 5b). In contrast, blocking antibody (VX15) significantly inhibited effects of SEMA4D and preserved EAAT-2 expression at near control levels. Anti-SEMA4D antibody (VX15) in the absence of exogenous SEMA4D had no effect on EAAT-2 expression. Effects were reproducible using an independent second source of primary human astrocytes.

To determine effects of SEMA4D on the astrocyte role in energy metabolism, we investigated expression of glucose transporter (GLUT-1) and monocarboxylate transporter (MCT) 4, the astrocytic transporter of lactate that facilitates shuttle to neurons. Significant reduction in expression of GLUT-1 and MCT4 on astrocytes was observed following incubation with recombinant SEMA4D (Fig. 5c). SEMA4D blocking antibody VX15 significantly inhibited these effects on GLUT-1 and MCT4 expression, while anti-SEMA4D antibody VX15 had no effect in the absence of SEMA4D ligand. Similarly, SEMA4D-induced reduction in GLUT-1 and MCT-4 levels was significantly inhibited by addition of PLXN-B1 and/or PLXN-B2 blocking antibodies (Fig. 5d).

We next investigated the functional ability of astrocytes to transport glucose in the presence of SEMA4D (Fig. 5e). SEMA4D reduced uptake of fluorescent non-metabolizable glucose analogs in cultured astrocytes within 24 h. Addition of SEMA4D blocking antibody VX15 at time 0 (solid lines) inhibited the SEMA4D-induced reduction of glucose uptake by astrocytes, while SEMA4D antibody VX15 alone in the absence of ligand had no effect. To determine if the effect of SEMA4D on glucose uptake is reversible, SEMA4D antibody was added after rSEMA4D treatment (*t* = 24 h, dotted lines) and effectively reversed the SEMA4D-induced conversion of astrocytes to a hypometabolic state.

We further characterized effects of SEMA4D on changes in astrocyte morphology, a hallmark of the reactive astrocyte phenotype. Purified astrocytes were cultured in the presence of recombinant SEMA4D, and the number and length of branches were quantified. Briefly, treated cells were examined at high resolution with apotome imaging configuration; resulting 3D-reconstructed images were semi-automatically quantified and projected in two-dimensions. For each treatment condition, over 100 DAPI-stained astrocytes were randomly picked for analysis. As an example, Additional File 3: Fig. S3b shows a slice (upper panel) of a selected astrocyte (red arrow) and lower panels show the integrated projection of the 3D image. Cells cultured in the presence of recombinant SEMA4D exhibited increased soma size, shortened primary branches and shorter and thicker fine processes (Fig. 5f, Additional File 3: Fig. S3b). Morphological changes were significantly inhibited by addition of SEMA4D blocking antibody.

Given the limitations of astrocyte culture systems, we sought to confirm in vitro effects of SEMA4D in a second species, primary rat cortical astrocyte cultures. Consistent with human astrocyte cultures, PLXN- B1 receptors are also highly expressed in rat astrocytes and recombinant SEMA4D triggers morphologic rearrangements, as evidenced by depolymerization of ~ 60% of F-actin, in comparison to control cultures (Additional File 4: Fig. S4a). Data were reproducible using primary rat astrocytes from two independent sources. To further characterize morphologic changes, we utilized a culture system that measures process extension into a cell-free area. In short, a plug is digested in the center of a confluent astrocyte layer to create a cell-free radius and this area is subsequently imaged to measure cellular coverage over time (Additional File 4: Fig. S4b). Recombinant SEMA4D (red open squares) was added at time 0 and compared to spontaneous extension (black) over a period of 20 h, at which point anti-SEMA4D (VX15, blue) or isotype control (HuIgG4, red closed squares) antibodies were added and incubated for an additional 28 h. Consistent with results in human astrocyte cultures, addition of SEMA4D reduces the extension of astrocyte cytoplasmic processes. This effect is, however, reversible and astrocytes recover the ability to extend projections following addition of SEMA4D blocking antibody.

Using several in vitro model systems, these results demonstrate consistent receptor-mediated effects of SEMA4D to induce changes associated with astrocyte reactivity, including downregulation of specific receptors and enzymes with associated loss of key astrocytic functions, as well as morphologic changes. Importantly, these changes were inhibited and reversed by blocking the binding of SEMA4D to its receptors.

## Discussion

**Fig. 6:**
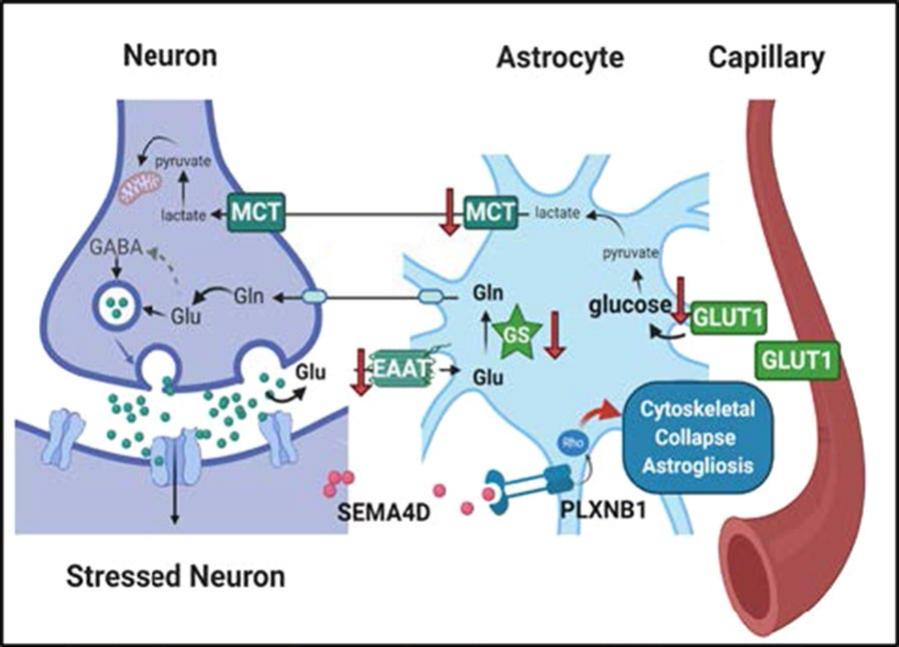
Schematic model for effects of SEMA4D on astrogliosis. In keeping with published data of others regarding changes in reactive astrocyte function, it is suggested that SEMA4D is upregulated in stressed neurons and binds to PLXN-B1/B2 receptors to trigger reactive astrocytes, characterized by morphologic reorganization of the cytoskeleton and retraction of dendritic processes, downregulation of glucose and lactate transporters (GLT1 and MCT), downregulation of glutamate receptor (EAAT2), and reduction in glutamine synthetase (GS), key functional transporters and enzymes in astrocytes. SEMA4D-induced reactive changes indicated by red arrows. Dysfunctional transport and conversion of metabolic and neurotransmitter substrates may reduce astrocytic neuroprotection mechanisms and impair recycling glutamate to glutamine in astrocytes and glutamine to glutamate and GABA in neurons. Image created with BioRender.com; adapted from Bélanger, Allaman, Magistretti. Cell Metab. 2011

Neuronal stress in slowly progressive neuroinflammatory and neurodegenerative diseases may be caused by a variety of physiological insults, including the accumulation of aggregated mutant huntingtin protein, amyloid, Tau and other misshaped proteins, but they each appear capable of inducing parallel pathogenic reactive responses. The role of astrocyte transformation in response to such stressors and their contribution to neuropathogenesis is complex. What mechanism(s) give rise to disease-associated pathogenic changes in astrocytes? The observations reported here suggest a model (Fig. 6) in which (i) SEMA4D is upregulated in response to stress at sites of neuronal injury; (ii) this triggers reactive changes in PLXN-B1/B2-positive astrocytes in close proximity to neurons; and (iii) compromises the normal role of astrocytes in glucose transport and glutamate recycling as well as potentially other deleterious changes due to loss or gain of function. Such changes, triggered by different stressors and injury, may be common to pathology in multiple chronic and slowly progressive neurologic diseases. A corollary is that blocking SEMA4D signaling could prevent or reduce disease-associated reactivity including loss of normal astrocyte functions.

We report here a novel finding, consistent across disease indications, that SEMA4D is upregulated predominantly in neurons during underlying disease progression in both HD and AD patients and disease models. We further provide evidence of the presence of reactive astrocytes characterized by increased GFAP immunoreactivity, downregulation of GS, and morphological changes associated with neurodegeneration and SEMA4D expression.

The association of SEMA4D signaling with disease progression was previously suggested by amelioration of disease symptoms and pathology following treatment of HD transgenic YAC 128 mice with SEMA4D blocking antibody [41]. We confirm here that treatment with SEMA4D blocking antibody also confers physiological and functional benefit in the CVN transgenic mouse model of AD. Others have previously reported that disruption of the GS-dependent conversion of glutamate to glutamine in reactive astrocytes has a differential effect on loss of inhibitory GABAergic relative to excitatory glutamatergic synapses [20]. NPY and somatostatin, produced mainly by GABAergic interneurons, are associated with cognitive and emotional processes, including learning and memory [59, 60]. Hippocampal NPY+ neurons are strongly affected in early stages of AD pathology and are significantly reduced in brains of AD patients [61] and in the CVN AD mouse model [56]. In the CVN model, treatment with SEMA4D blocking antibodies prevented astrocytic transformation to a reactive phenotype, as reflected in fractal dimension analysis and preservation of GS expression. SEMA4D blockade also prevented the characteristic loss of inhibitory NPY+ and somatostatin + neurons in this disease model. Importantly, antibody treatment ameliorated cognitive deficits associated with spatial memory and learning in the CVN AD model as reflected in the radial arm water maze test. The CVN mouse model is considered appropriate for this study because the background APP variant expressed in this model is associated with a molecular subtype of AD that appears to be driven by inflammatory processes [57]. We have not investigated the effect of SEMA4D blockade in AD models in which other neurodegenerative factors predominate. However, as we report elsewhere [62], there is neuronal upregulation of SEMA4D and striking physiological benefits to SEMA4D blockade in the Mecp2^T158A/y^ mouse model of Rett syndrome. Rett syndrome is considered a neurodevelopmental rather than neurodegenerative disease, but it appears that there are overlapping, inflammation related, pathogenic mechanisms.

The dramatic upregulation of SEMA4D in brain tissue in different neurological diseases, together with consistent evidence of reactive astrocytes expressing PLXN receptors established a connection that led us to hypothesize that the ligand may be responsible for triggering cytoskeletal and reactive changes via receptor binding on astrocytes. In the brain, astrocytes express the most abundant levels of PLXN-B1 and PLXN-B2 receptors and have been shown to respond to SEMA4D signaling, which can induce cytoskeletal and inflammatory alterations [39]. Interestingly, in Q175 HD mice and human HD, Diaz et al. [9] reported that cytoskeleton-related genes were among the top upregulated genes in astrocytes early in disease and that significant astrocyte reactivity can be found in the striatum. We demonstrate here that astrocytes express both PLXN-B1 and -B2 receptors for SEMA4D and that in purified astrocyte cultures recombinant SEMA4D induces not only morphologic changes, but, importantly, other changes related to astrocyte function, including downregulation of GLUT-1 and MCT-4 transporters required for metabolic functions, and EAAT-2 transporter essential for neurotransmitter recycling. These SEMA4D-induced effects on astrocyte morphology and downregulation of key metabolic transporters and enzymes were prevented by SEMA4D and PLXN-B1/B2 blocking antibodies. Importantly, inhibition of glucose transport by recombinant SEMA4D could be reversed by neutralizing antibody after 24 h. These observations support the therapeutic benefit of antibody blockade in animal disease models of HD, AD and Rett syndrome and suggested that a measure of brain metabolic activity, such as FDG-PET imaging [24], could serve as a potential biomarker of drug effect in clinical trials.

We provide here evidence of SEMA4D regulation of astrocytic function, but it is important to note that there are other potential effects of SEMA4D blockade including an impact on crosstalk between astrocytes and other glia that may contribute to neuroprotective benefits. We previously reported that SEMA4D blocking antibody prevents inflammatory activation of murine Iba-1 + microglia at the site of demyelinated lesions in spinal cord [32]. While microglia were not the focus of this report, their potentially important contributions to disease pathology and extensive crosstalk among glia warrant further investigation in the setting of neurodegenerative disease. Recently, Clark et al. [39] reported that PLXN-B1/B2-positive astrocytes can interact directly with a subset of SEMA4D positive microglia in the context of CNS inflammation in EAE. Additional mechanisms may contribute to the role of SEMA4D in disease pathology. We have previously reported that SEMA4D inhibits the migration and differentiation of glial progenitor cells that are able to replenish astrocytes and repair demyelinated lesions [32]. Blocking SEMA4D could, therefore, also change the balance between normal and inflammatory glia as well as between normal and compromised myelinated neurons. Other reported effects of blocking SEMA4D include protecting and restoring the integrity of the blood–brain barrier [32] which could also have a profound impact on transport, tissue environment, and synaptic activity.

There are several limitations to this study. First, the study reports a broad survey of SEMA4D expression within diseased brain tissue; more detailed analyses, e.g., single-cell gene expression analysis in affected regions and among cellular subtypes may further elucidate patterns of receptor and ligand expression, related signaling pathways, and mechanisms of neuronal stress and astrocyte reactivity. Additionally, in vitro astrocyte cultures pose inherent limitations related to cell isolation and artificial culture conditions. To mitigate these limitations, results were reproduced in cells from different sources and two different species, culture conditions employed specially formulated growth media, and reported data are restricted to analysis of GFAP+ cells. Nonetheless, the possible contribution of low-level contaminants of microglial or endothelial origin must be acknowledged. Alternate in vitro systems, such as use of cells derived from diseased individuals, co-cultures or brain slice models could be informative to more fully characterize the effects of neuronally expressed SEMA4D on astrocytes and interaction with other PLXN-B1/-B2+ cells in the microenvironment, and potential downstream effects on neuronal function.

## Conclusion

Collectively, these findings support the therapeutic potential of SEMA4D antibody blockade to regulate glial cell function and reduce neuronal toxicity and dysfunction in neurodegenerative diseases including HD and AD. Studies of neurodegenerative diseases have long focused on neurotoxic proteins, however reactive astrocytes are increasingly recognized for their important roles in disease progression [17]. Strategies that target these pathogenic processes have the potential to be broadly applicable to multiple devastating neurologic diseases. Clinical evaluation of the safety and efficacy of pepinemab (VX15/2503), a humanized anti-SEMA4D monoclonal antibody, has been initiated, including a completed Phase I trial in patients with multiple sclerosis (NCT01764737) [63], a Phase II trial in Huntington’s disease (NCT02481674) that is complete and will be reported elsewhere [64], and an ongoing Phase I/II study of pepinemab in Alzheimer’s disease (NCT04381468). Clinical and imaging outcomes including FDG-PET may provide further evidence of the biologic activity of astrocytes and their contribution to disease progression.

## Supplementary Information

**Additional File 1: Fig. S1.** Sema4D Is Expressed On Olig2-Positive Oligodendrocytes Of Dentate Gyrus Regions In HD Mice.

**Additional File 2: Fig. S2.** SEMA4D, NeuN+ neuronal density and latency errors in Radial Arm Water Maze in AD mice. (PDF 81 KB)

**Additional File 3: Fig. S3.** GFAP and morphologic assessments in human astrocyte cultures.

**Additional File 4: Fig. S4 and methods**. PLXN-B1 expression and morphologic assessments in rat astrocyte cultures.

## Data Availability

All data are available in the main text or the supplementary materials.

## Abbreviations

SEMA4D: Semaphorin 4D
HD: Huntington’s disease
AD: Alzheimer’s disease
GFAP: Glial fibrillary acidic protein
PLXN: Plexin
BBB: Blood–brain barrier
KI: Knock-in
WT: Wild type
SEM: Standard error of mean
Df: Fractal dimensions
GS: Glutamine synthetase
M: Male
F: Female
