# Supplementary material for: Semaphorin 4D is upregulated in neurons of diseased brains and triggers astrocyte reactivity
Source: J Neuroinflammation. 2022 Aug 6;19:200. doi: 10.1186/s12974-022-02509-8 (PMC9356477; doi:10.1186/s12974-022-02509-8)
Supplement: Supplementary file 1 — Additional File 1: Fig. S1. Sema4D Is Expressed On Olig2-Positive Oligodendrocytes Of Dentate Gyrus Regions In HD Mice. [file 12974_2022_2509_MOESM1_ESM.pdf]

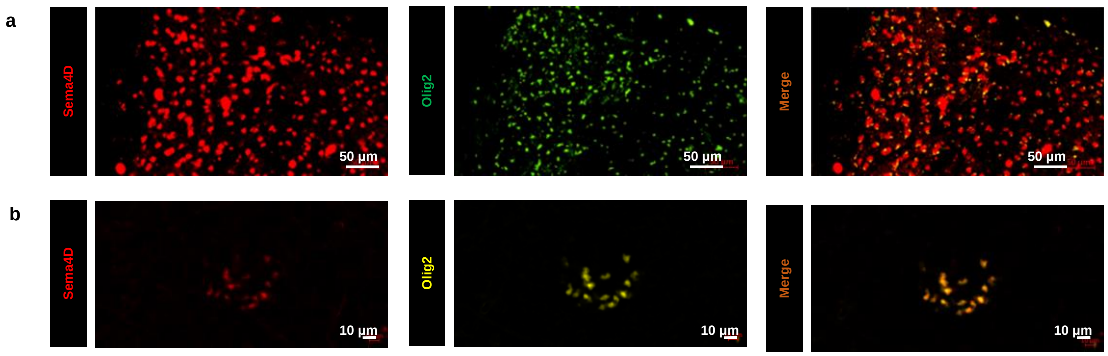

Additional file 1. Fig. S1: SEMA4D is expressed on Olig2-positive oligodendrocytes of dentate gyrus regions in HD mice at 9.3 months of age. A. Plan-Apochromat 40x/0.95 objective SEMA4D is expressed on both oligodendrocytes (SEMA4D+Oligo2+, yellow) and the closely associated neurons (SEMA4D+ but Olig2-, red. See also Fig. 1). **B.** Higher magnification images of SEMA4D+ oligodendrocytes: Plan-Apochromat 63x/1.4, oil objective and ApoTome system (Carl Zeiss) with Axiocam 702 Monochrome Camera.
