# Supplementary material for: Semaphorin 4D is upregulated in neurons of diseased brains and triggers astrocyte reactivity
Source: J Neuroinflammation. 2022 Aug 6;19:200. doi: 10.1186/s12974-022-02509-8 (PMC9356477; doi:10.1186/s12974-022-02509-8)
Supplement: Supplementary file 2 — Additional File 2: Fig. S2. SEMA4D, NeuN+ neuronal density and latency errors in Radial Arm Water Maze in AD mice. (PDF 81 KB) [file 12974_2022_2509_MOESM2_ESM.pdf]

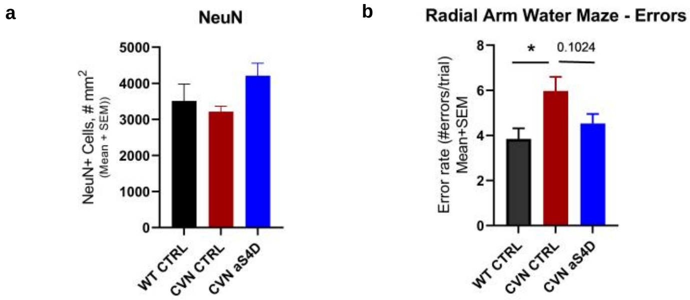

Additional file 2. Fig. S2: CVN and WT mice were treated *in vivo* with mouse anti-SEMA4D "aS4D" or mouse isotype-matched control antibody"CTRL" Brains were collected at week 41. **a**. Hippocampal region CA1-3 including stratum oriens, radiatum and pyramidal layer of CVN and wild type mice were stained for NeuN. Differences between groups were not statistically significant, as determined by 2-way ANOVA with Bonferroni's adjustment. **b**. Error rate in Radial Arm Water Maze at week 36, group mean of all animals+SEM is shown. Statistical significance was determined by unpaired t-test, p-value is indicated; * $p<0.05$.
