# Supplementary material for: Semaphorin 4D is upregulated in neurons of diseased brains and triggers astrocyte reactivity
Source: J Neuroinflammation. 2022 Aug 6;19:200. doi: 10.1186/s12974-022-02509-8 (PMC9356477; doi:10.1186/s12974-022-02509-8)
Supplement: Supplementary file 3 — Additional File 3: Fig. S3. GFAP and morphologic assessments in human astrocyte cultures. [file 12974_2022_2509_MOESM3_ESM.pdf]

a

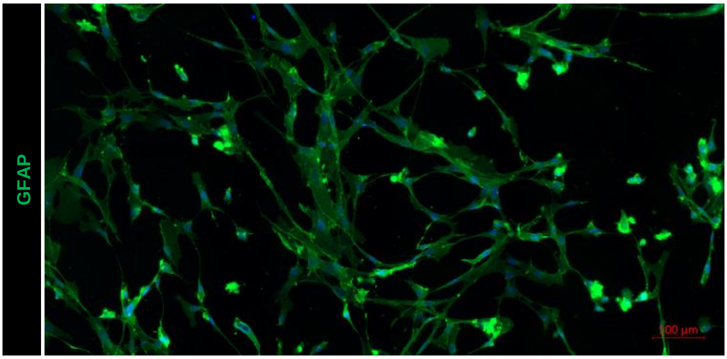

b

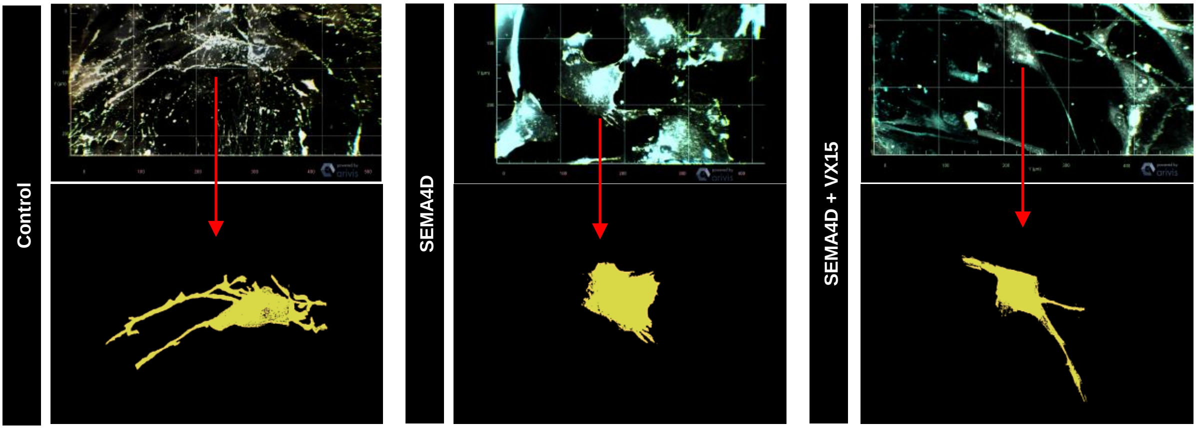

Additional file 3. Fig. S3: a. GFAP in purified human astrocyte cultures (GFAP, green; DAPI, blue)**. b.** Human primary astrocytes were cultured in presence of rSEMA4D or control protein. Antibody blocking effect was determined by incubation with rSEMA4D or control protein (5 ug/ml), in presence/absence of anti-SEMA4D antibody/VX15 or isotype control human IgG4 antibody (25 ug/ml) for 48 hours; each condition was assessed in 6 replicate wells. Morphologic changes from 3D reconstruction micrographs showing differences in soma size and length and number of primary processes. Representative images are shown; each square represents area of 100X100 μm^2 (upper panels) from one stack of the 3D image, and local connected fractal dimension (LCFD) characteristics from the integrated projection of the 3D image (lower panels) of one representative astrocyte (indicated by arrows to lower panels).
