# Supplementary material for: Semaphorin 4D is upregulated in neurons of diseased brains and triggers astrocyte reactivity
Source: J Neuroinflammation. 2022 Aug 6;19:200. doi: 10.1186/s12974-022-02509-8 (PMC9356477; doi:10.1186/s12974-022-02509-8)
Supplement: Supplementary file 4 — Additional File 4: Fig. S4 and methods. PLXN-B1 expression and morphologic assessments in rat astrocyte cultures. [file 12974_2022_2509_MOESM4_ESM.pdf]

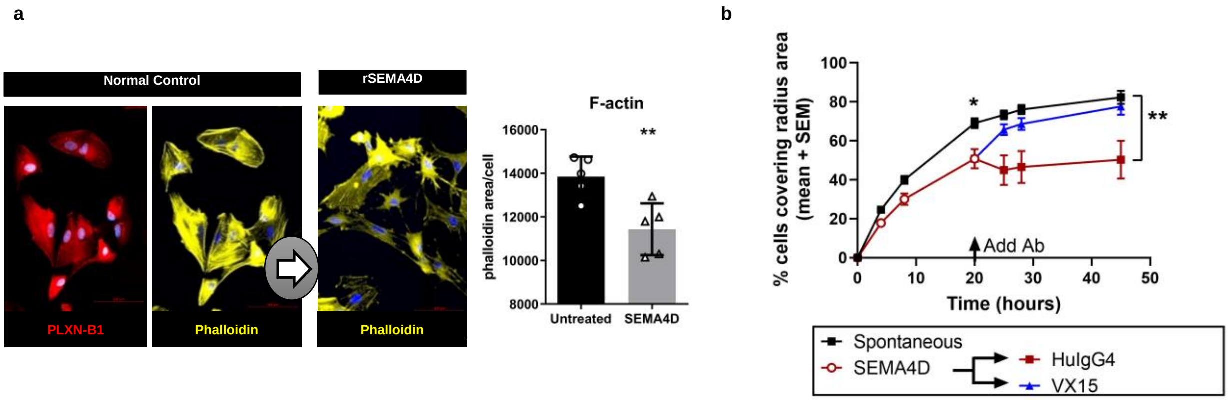

Additional file 4. Fig.S4: a. Primary rat astrocyte cultures were stained for expression of receptor PLXN-B1 (red), F-actin filaments (phalloidin, yellow) and nuclei (DAPI, blue) before (center) and after (right) one-hour treatment with recombinant SEMA4D. Representative images are shown. Mean phalloidin-positive area/cell in a field of ~ 300 GFAP+ astrocytes was quantified using ImagePro software in each of five separate culture wells/condition. **b**. SEMA4D inhibits process extension of astrocytes; pepinemab reverses effects. Astrocyte process extension into cell-free area was determined following culture of purified rat astrocytes for the indicated time in the presence or absence of recombinant SEMA4D (15 mcg/ml), added at time 0. SEMA4D antibody “VX15” or isotype control antibody “HuIgG4” (50 ug/ml) was added at time = 20 hours to determine whether the effect is reversible. Results in replicate wells (n=6) at each time point are normalized to cell-free area at time 0. Statistical significance was determined using two-way ANOVA
